# Design of Ti-Mo-W Alloys and Its Correlation with Corrosion Resistance in Simulated Body Fluid (SBF)

**DOI:** 10.3390/ma16062453

**Published:** 2023-03-19

**Authors:** Ştefan-Ioan Ghica, Valeriu-Gabriel Ghica, Mircea-Ionuţ Petrescu, Gheorghe Iacob, Victor Geantă, Mihai Buzatu, Elena Ungureanu

**Affiliations:** Department of Engineering and Management of Obtaining Metallic Materials, University Politehnica of Bucharest, 313 Splaiul Independentei, J Building, RO-060042 Bucharest, Romania; ghica_stefan@yahoo.com (Ş.-I.G.); gvghica@yahoo.com (V.-G.G.); ipetrescu@yahoo.com (M.-I.P.); victor.geanta@upb.ro (V.G.); mbuzaturo@yahoo.com (M.B.); elena.ungureanu1102@upb.ro (E.U.)

**Keywords:** Ti-Mo-W alloys design, molecular orbital calculation, bond order, metal—orbital energy level, SEM-EDS, corrosion resistance, simulated body fluid

## Abstract

Titanium and its based alloys are frequently selected for designing biomedical implants and it is thus necessary to study as detailed as possible their corrosion behavior in biological solutions, such as those in the human body environment. In this paper, with the use of molecular orbital calculation, we designed and developed alloys in the Ti-19Mo-xW system (x = 7, 8, 9, and 10 wt%) and investigated the influence of different contents of tungsten on the behavior of Ti-19Mo-xW alloy samples following corrosion in simulated body fluid (SBF). The values of $\bar{Bo}$ (bond order) and $\bar{Md}$ (the metal—orbital energy level) were calculated for each alloy and correlations were established between $\bar{Bo}$ and the content of tungsten. It was found that with the increase in tungsten content, the value of $\bar{Bo}$ increases. Regarding the values of the corrosion resistance in SBF that resulted from the investigated alloys, the Ti19Mo7W alloy is distinguished by the lowest value of the corrosion current density and the lowest corrosion rate.

## 1. Introduction

Titanium alloys were developed for biomedical applications (over 70% of the devices used) with remarkable properties in the field of biocompatible materials, namely, superior biocompatibility, better corrosion resistance and specific strength, low Young’s modulus of elasticity, and the fact that it does not cause allergic problems [1,2].

From the entire range of biocompatible titanium-based alloys, the Ti-6Al-4V alloy stands out, being very often used in medical applications (such as orthopedic implants). However, as reported in many studies, it presents a health risk. Due to the release of toxic Al and V ions, this can cause cytotoxicity. These elements are considered as toxic for the body, leading to repercussions over time (neurological and respiratory disorders) [3,4,5,6,7].

The concentrations of chloride ions (Cl^−^) or proteins that act as electrolytes and lead to a low oxygen content in the body fluid could initiate and support the corrosive process. This contributes to a lower or greater extent to the release of these toxic elements, and when they accumulate in significant quantities, they can cause allergenic or even carcinogenic effects [8,9,10,11,12,13]. To avoid these undesirable aspects, many researchers have published studies on new types of titanium-based alloys, which replaced the problematic elements with others that can lead to the same characteristic properties, and developed alloys with a low Young’s modulus with β or near β phase [2,6,12,14,15,16,17].

Currently, when designing titanium alloys, non-toxic elements for the human body with good biocompatible characteristics, are considered. Elements, such as Niobium, Tantalum, Zirconium, Molybdenum, and Tungsten form beta phases with Titanium, which then form different alloy systems that overcome the toxicity associated with Ti-6Al-4V alloys—Ti-Nb-Zr, Ti-Nb-Zr-Sn, Ti-Nb-Ta-Zr, Ti-Mo-Si, Ti-Mo-Zr-Ta, Ti-Mo-Zr-Fe [18,19,20,21,22,23,24]. In [13], two new titanium alloys—Ti20Mo7Zr and Ti20Mo7Zr0.5Si—were studied in terms of mechanical characteristics and electrochemical behavior. The electrochemical studies were carried out in a simulated body fluid (SBF) and saline medium, and the conditions for fever were even simulated. The results indicated that by lowering the concentration of silicon in the obtained samples, the values for corrosion resistance increase.

The Ti-15%Mo alloy appeared in the 1950s. Considered as a corrosion-resistant titanium alloy [3], it was proposed to replace nickel-based superalloys, and has been used to create pipes for transporting corrosive liquids in chemical plants, as well as biocompatible materials. The high price, low tensile strength, and low tribological properties were impediments overcome with difficulty. The Ti-Mo alloy belongs to the class of beta-titanium alloys. These alloys have a low modulus of elasticity, high resistance to corrosion, and are well tolerated in the human organism. The stable beta structure at room temperature is obtained by a correct choice of the chemical composition and the applied thermal treatments. Tungsten is one of the stabilizing beta-Ti elements, in addition to Mo, V, Nb, Hf, Ta, Fe, Cr, Mn, etc. In most studies, Ti15Mo was chosen as the base alloy, whereas in recent research [25], two alloys were studied—Ti80Mo10W10, Ti90Mo5W5— which were obtained by mechanical alloying for 20 h and conventional sintering.

The alloys investigated in this paper, which continue a series of research related to alloys in the Ti-Mo-W system [26,27,28,29,30,31], can be a solution to be considered for developing and designing implants that will serve for a longer period. In addition, they can assist in avoiding possible health problems caused by the Al and V content of classic titanium alloys, while maintaining the superior characteristics of the titanium alloys quite well-known, especially the superior corrosion resistance in biological environment that is excellently combined with high mechanical properties.

When designing alloys, several parameters can be considered: The bond order ($\bar{Bo}$), a measure of the strength of the covalent bond between the M and X atom, the energy level of the d orbital ($\bar{Md}$), the ratio between valence electrons and atom (*e/a*), and the molybdenum equivalent (the $\bar{Mo}$ equivalency). The theory of d-electron alloy design is now widely used in the attainment of titanium alloys with a lower Young’s modulus and good corrosion resistance in simulated biological solution (SBF) [32,33].

The addition of a sufficient amount of Molybdenum gives Titanium a very good resistance to corrosion [31,34]. In several studies [2,16,35,36], it is observed that a minimum percentage of 15 wt% Mo can lead to an increase in corrosion resistance in biological environments at high temperatures for titanium alloys [8,31,36]. All these studies are developed following the design of alloys through theoretical methods without any trial-and-error experiments (a molecular orbital approach to alloy design) that involve the use and combining of the parameters mentioned earlier: $\bar{Bo}$ and $\bar{Md}$ [37,38,39]. The energy level controls the direction of charge transfer, namely, it is related to electronegativity. The element with higher electronegativity has the lower $\bar{Md}$ energy level [40].

As the electronegativity increases from W to Mo, it follows that $\bar{Md}$ is lower. The values of $\bar{Bo}$ and $\bar{Md}$ in bcc titanium are for Ti: $\bar{Bo}$ = 2.790, Md (eV) = 2.447 (3d); for Mo: $\bar{Bo}$ = 3.063, Md (EV) = 1.961 (4d); and for W: $\bar{Bo}$ = 3.125, Md (eV) = 2.072 (5d) [41]. It was demonstrated that the alloy with superior $\bar{Bo}$ has higher corrosion resistance.

**Figure 1:**
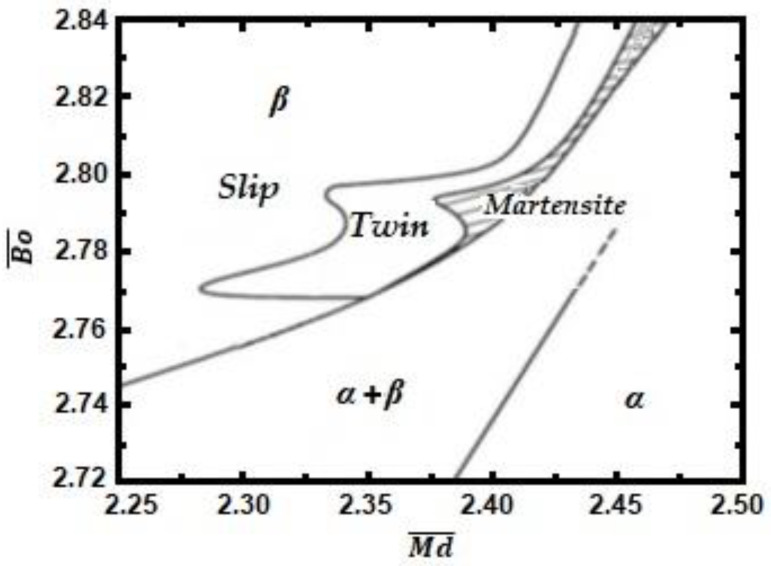
The $\bar{Bo}$ and $\bar{Md}$ stability-phase map, showing the positions of the designed alloys [38,39].

The aim of the study is to investigate the alloying effect of Mo and W concerning the stability of bcc—β-Ti phase. In previous studies, we investigated the biocompatible alloys from the Ti-15Mo-xW (1–11% mass) system [26,27,28,29,30,31]. In the present work, we increased the Mo concentration to 19% and varied the W concentration between 7 and 10%. However, these compositions lead to close values of $\bar{Bo}$ and $\bar{Md}$ ($\bar{Bo}$ = 2.82; $\bar{Md\;}$= 2.39), as in the case of the previously studied alloys (see $\bar{Bo}$ and $\bar{Md}$ stability-phase map in Figure 1). They all position themselves in the beta stable domain, and data are confirmed by other researchers [2,6,12,14,15,16,17].

The study started with theoretical calculations obtained by the molecular orbital calculation of $\bar{Bo}$ and $\bar{Md}$. The increase in the Mo content (to 19 wt% compared to the established value of 15 wt%) and its correlation with the increase in the W content from 7 to 10 wt% aimed to direct the alloy into the stable beta range. The calculated data are in accordance with those of [40], and were correlated with the results of corrosion tests in simulated body fluid (SBF) by means of linear polarization technique. Tungsten and molybdenum have a higher solubility than titanium. This is primarily due to the close atomic radius values of the elements. Its values are for: Titanium—0.176 nm, Molybdenum—0.190 nm, and Tungsten—0.193 nm. Second, it is due to the similar crystal structure (bcc) and close electronegativity values: Ti—1.54, Mo— 2.16 and W—2.36 [26].

## 2. Materials and Methods

### 2.1. Sample Preparation

**Figure 2:**
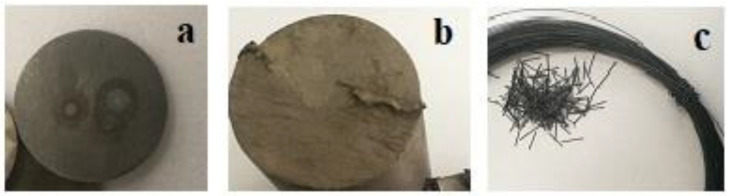
Materials used in the VAR process for obtaining Ti-19Mo-xW samples. Materials used in the VAR process for obtaining Ti-19Mo-xW samples: (**a**) Titanium; (**b**) Molybdenum; (**c**) Tungsten.

The materials used in the attainment of Ti-19Mo-xW (x = 7, 8, 9, and 10 wt%) were the following: Titanium of commercial purity—Ti grade 1 (ASTM B265 G1), commercial purity Molybdenum (ASTM B467-GrMo-1), and commercial purity Tungsten W1 (ASTM B348 GrW1) in the shape of φ 0.5 mm wire. The macroscopical images of materials used to obtain samples from Ti-19Mo-xW alloys are shown in Figure 2.

**Figure 3:**
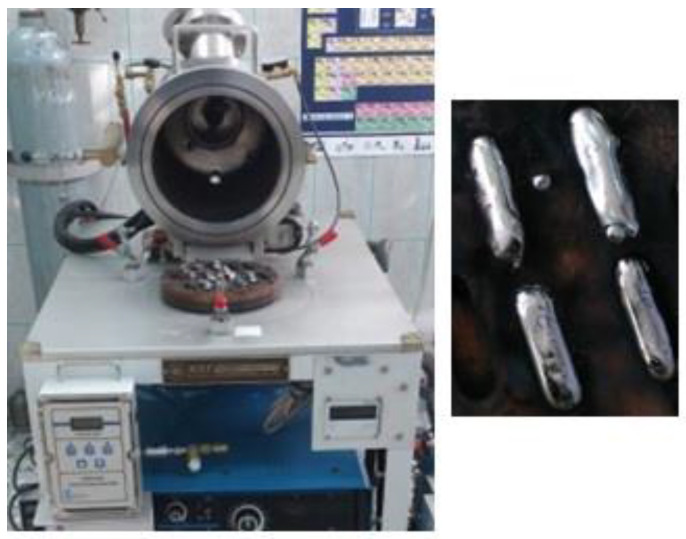
VAR furnace used in the experiments and the samples obtained.

The attainment of the alloys sample through successive melting and remelting operations took place in a Tungsten (anode) electrode arc furnace (VAR model MRF ABJ-900—Materials Research Furnaces, Inc., Suncook, NH, USA); all the samples were positioned in a copper melting crucible (cathode) cooled with water, as seen in Figure 3. The temperature attained in this type of furnace is beyond 3500°C, sufficiently high for the melting of the chosen alloys (melting and mixing), which contains metals with a high melting point (Ti—1668 °C; Mo—2623 °C; W—3422 °C) [26,27,28,29]. 

The chemical compositions of the metals used in the experimental research are presented in Table 1.

As noted in the previous work [31], it was found that the best results after corrosion testing were for the Ti-15Mo-5W alloy. The problem for this system of alloys is that a good homogeneity is very difficult to achieve within the limits imposed by the VAR method (remelting is necessary) and the elements of the chemical composition. The melting point of Tungsten was close to the maximum heat capacity of the furnace, and thus two successive melts were performed. As a result, we tried to expand the compositional field, without worsening the properties of the alloy and maintaining a similar ratio between the elements. The 15Mo/5W ratio is 3, close to the 19Mo/7W ratio of 2.71.

### 2.2. Sample Characterization

The microstructural images of the samples were obtained by scanning electron microscopy (SEM) using the Quanta Inspect F50 microscope (Thermo Fisher Scientific Inc., Waltham, MA, USA) with the following technical specifications: A high-resolution field-emission electron microscopy through the thermal Schottky effect, resolution of 1 kV (3.0 nm without BD) to 30 kV, acceleration voltage: 0.2–30 KV, and maximum beam current of 1.0 nm (200 nA). The elemental analysis of samples (wt%) was determined analytically by energy-dispersive X-ray spectrometry (EDX). To observe the distribution of elements in the base metal, energy-dispersive spectroscopy (EDS) mapping was performed. This was applied for the first and last alloy sample.

**Figure 4:**
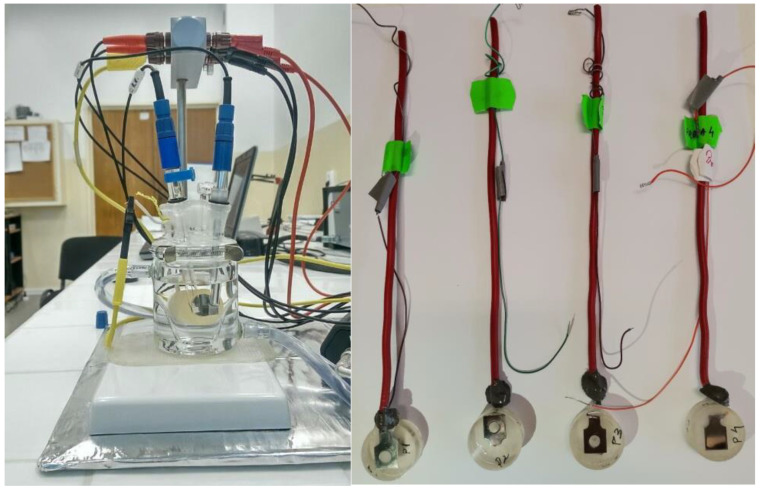
PARSTAT 4000 installation [31] (**left**) and the samples after the corrosion tests (**right**).

Corrosion resistance tests were performed using the PARSTAT 4000 installation (Princeton Applied Research, Oak Ridge, TN, USA), as seen in Figure 4 left. Electrochemical tests on the Ti-19Mo-xW samples (Figure 4 right) were carried out by the linear polarization technique according to ASTM G5–94 (2011) in simulated body fluid solution. 

The calculation of the polarization resistance was carried out according to ASTM G59-97 (2014) [42] using the Stern-Geary equation:

(1) $$R_{p}=\frac{1}{2.3}\frac{\beta_{a}|\beta_{c}|}{\beta_{a}+\left|\beta_{c}\right|}\frac{1}{i_{corr}}$$

where:

*β_a_*= anodic Tafel slope;
*β_c_* = cathodic Tafel slope;
*i_corr_* = corrosion current density, [μA/cm^2^].

The calculation of the corrosion rate was according to ASTM G102-89 (2015) [43] using the following equation:

(2) $$CR=K_{i}\frac{i_{corr}}{\rho }EW$$

where:

*CR* = corrosion rate;
*K_i_* = constant which defines the units of corrosion rate (3.27 × 10^−3^);
*ρ* = density [m/V]; *i_corr_*—corrosion current density, [μA/cm^2^];
*EW* = equivalent weight.

The SBF solution had a pH of 7.4 at the human body temperature (37 ± 0.5 °C) and was heated and recirculated using the bath model CW-05G produced by Jeio Tech [30]. The composition of SBF was: 8.035 gL^−1^ NaCl, 0.350 gL^−1^ NaHCO_3_^−1^, 0.225 gL^−1^ KCl, 0.231 gL^−1^ K_2_HPO_4_·3H_2_O, 0.311 gL^−1^ MgCl_2_·6H_2_O, 39 mL^−1^ 1 M-HCl, 0.292 gL^−1^ CaCl_2_, 0.072 gL^−1^ Na_2_SO_4_, 6.118 gL^−1^ (CH_2_OH)_3_CNH_2_.

## 3. Results and Discussion

### 3.1. Compositional Evaluation

The samples of cast ternary Ti-19Mo-xW alloys (x = 7, 8,9, and 10 wt% W) were micro-compositionally analyzed using an EDS spectrometer showing the elements present in the analyzed area, and this can help us in estimating their relative abundance. 

The results of the elemental analysis (in wt%) of the Ti-19Mo-xW alloys used in the experimental research were presented in Table 2 (Code 1 to 4) as well as for the Ti6Al4V alloy used for comparison (Code 5). The EDS characterization for the Ti-19Mo-xW alloy samples is presented later, in 3.3—Morphological Evaluation, together with the SEM image of each sample (Code 1 to 4).

### 3.2. Molecular Orbital Calculation of Ti-Mo-W Alloys

The theory of d-electron alloy design is currently used for the designing of titanium alloys with a lower Young’s modulus and good corrosion resistance in simulated biological solution (SBF). The influence of tungsten addition on $\bar{Bo}$ and $\bar{Md}$ values are calculated using Equations (3) and (4) and are presented in Table 3.

(3) $$\bar{Bo}=\sum Bo_{i}x_{i},$$

(4) $$\bar{Md}=\sum Md_{i}x_{i},$$

where:

*Bo_i_* = bond order for element *i*;
$x_{i}$ = at % of element *i*;
*Md_i_* = the energy level of the d orbital for the element *i*.

The values calculated and presented in Table 3 position the obtained alloys in the bcc—titanium domain, which could be compared with the values in Figure 1 [39].

With the growth of the W content in the investigated alloys of the Ti-19Mo-xW type, the increase in the $\bar{Bo}$ parameter is observed (from the value of 2.8281 for a content of 7 wt% W to 2.8315 in the case of the addition of 9 wt% W).

If the value of $\bar{Bo}$ increases, the chemical bond between the atoms of the component elements for the investigated alloy becomes stronger, and as the electronegativity increases, the value of the $\bar{Md}$ parameter decreases. The bond strengths correlate with the electronegativity difference. These results are in accordance with those of other authors [38] and presented in Figure 1, proving that the obtained alloys are in the beta phase stability region. 

### 3.3. Morphological Evaluation

**Figure 5:**
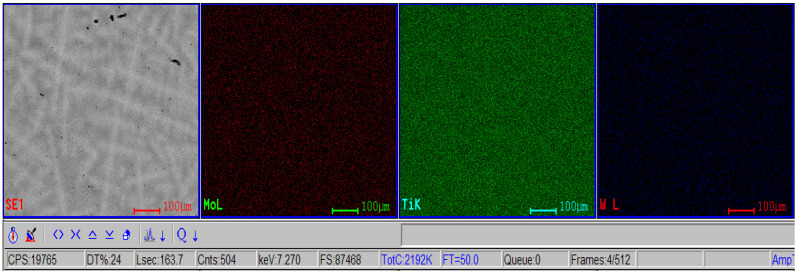
SEI image and BSED mapping of the Ti19Mo7W sample (Code 1) showing the repartition of W, Mo, and Ti.

**Figure 6:**
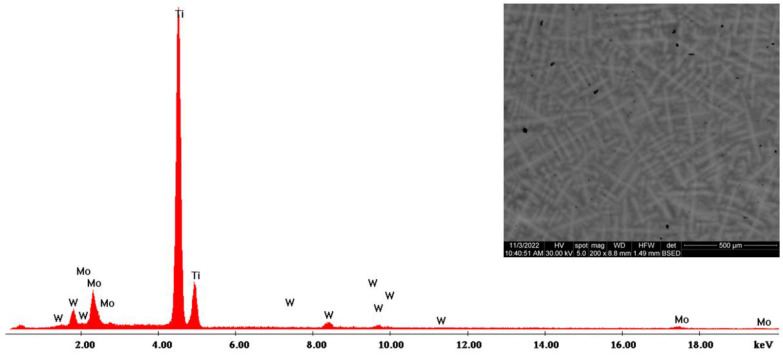
SEM—EDS characterization of Ti19Mo7W alloy (Code 1).

**Figure 7:**
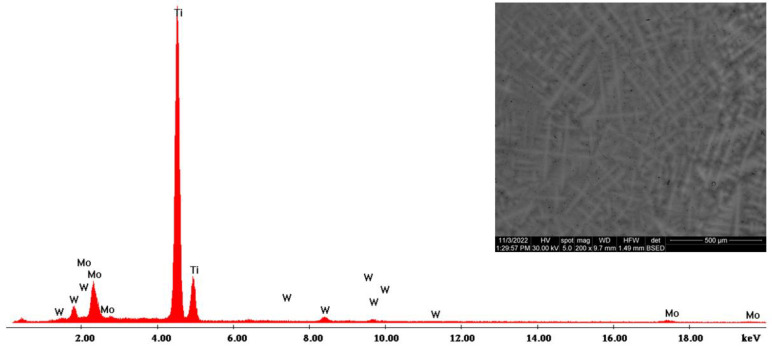
SEM—EDS characterization of Ti19Mo8W alloy (Code 2).

**Figure 8:**
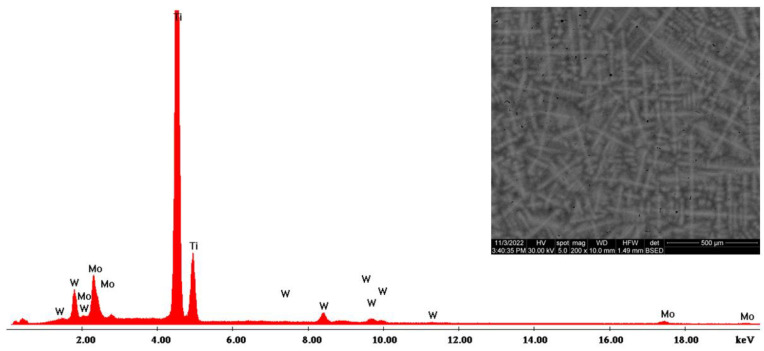
SEM—EDS characterization of Ti19Mo9W alloy (Code 3).

**Figure 9:**
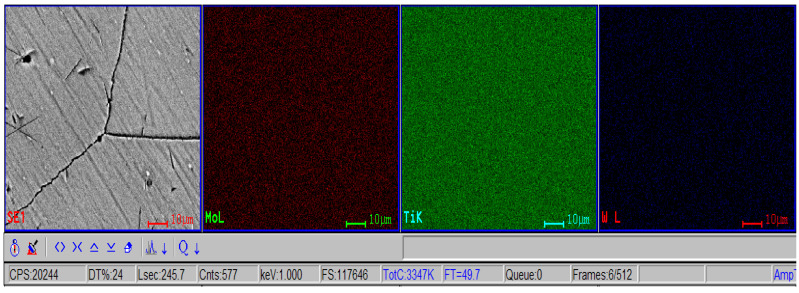
SEI image and BSED mapping of the Ti19Mo10W sample (Code 4) showing the repartition of W, Mo, and Ti.

**Figure 10:**
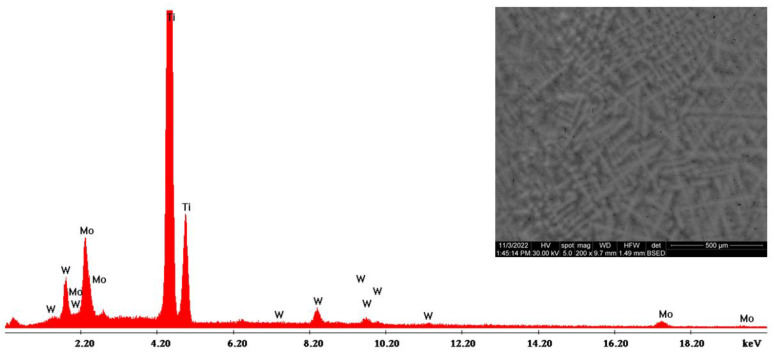
SEM—EDS characterization of Ti19Mo10W alloy (Code 4).

The morphological evaluation carried out in this study is shown in Figure 5, Figure 6, Figure 7, Figure 8, Figure 9 and Figure 10. For all Ti-19Mo-xW alloys (Code 1 to 4) was performed a SEM-EDS characterization (Figure 6, Figure 7, Figure 8 and Figure 10), and for two samples (Code 1—Ti19Mo7W and Code 4—Ti19Mo10W), a BSED analysis was also carried out (Figure 5 and Figure 9). We can observe that there is a uniform distribution of the element’s molybdenum and tungsten in the titanium structure after the successive melting, as indicated by the BSED mapping and SEI image of the Ti19Mo7W in Figure 5 and Ti19Mo10W in Figure 9. The SEM images show the formation of a dendritic structure for all the obtained samples. This is also due to the faster cooling of the samples that were melted and maintained for solidification in the water-cooled copper crucible. Electron microscopy (SEM and EDS) was used to evidence the uniform distribution of the alloying elements after the second melting.

### 3.4. Corrosion Resistance Assessment

The corrosion resistance assessment of Ti-19Mo-xW alloys (x = 7, 8, 9, and 10 wt%) was performed by linear polarization electrochemical tests. After testing, the corrosion rate of samples was quantitatively determined (Code 1 to 4). The samples were exposed to SBF media to evaluate the corrosion behavior of the investigated Ti-19Mo-xW alloys, and the main electrochemical parameters were extracted. To assess the corrosion rate for the Ti-19Mo-xW alloys, the polarization resistance method was used, through which the corrosion current density and the corrosion potential can be obtained.

**Figure 11:**
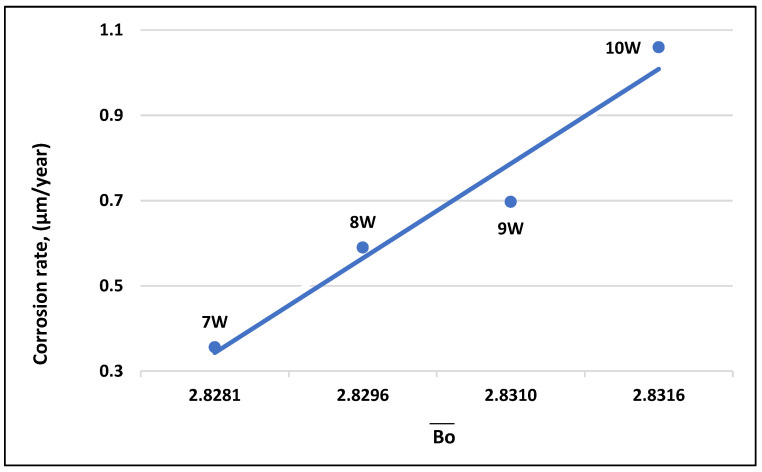
Correlation of corrosion rate, *CR* (µm/year) with Bo for beta Ti-19Mo-xW alloys.

Based on the results obtained from the polarization curve and electrochemical parameters of the corrosion process, the Ti-19Mo-xW alloys were shown to be corrosion resistant. Table 4 presents the main electrochemical parameters obtained from the corrosion tests performed in SBF. Figure 11 shows the correlation between the corrosion rates of the investigated alloys, depending on the $\bar{Bo}$ parameter values.

Regarding the corrosion potential (*E_cor_*), a more electropositive value of the *E_cor_* corrosion potential denotes a more “noble” character from an electrochemical viewpoint. Therefore, from this perspective, the Ti6Al4V alloy holds the most electropositive value (−186.2 mV).

According to literature data, a good corrosion resistance is provided by a low corrosion current density (*i_corr_*). Taking this criterion into account, we can observe that the titanium-based alloy containing W in proportion of 7 wt%, which registers the lowest value (29.214 nA/cm^2^), demonstrates that it presents a higher resistance to corrosion in comparison to the other investigated alloys. The current densities recorded for the rest of the Ti-based alloys containing 8, 9, and 10 wt% W, have higher values than the reference alloy (Ti6Al4V).

After calculating the corrosion rate (*CR*) of the alloys following the electrochemical tests performed in SBF, it is observed that the lowest value is obtained for the alloy containing 7% W (0.306 µm/year) followed by the value obtained in the case of the reference sample—Ti6Al4V (0.322 µm/year).

Of these, sample 4 with 10 wt% W (Figure 11) shows the highest corrosion rate value partly since Tungsten is resistant to atmospheric corrosion, but reacts at room temperature with halogens (Fluorine), and the SBF test solutions contain halogens. If the W content increases, the corrosion rate will also be higher.

**Figure 12:**
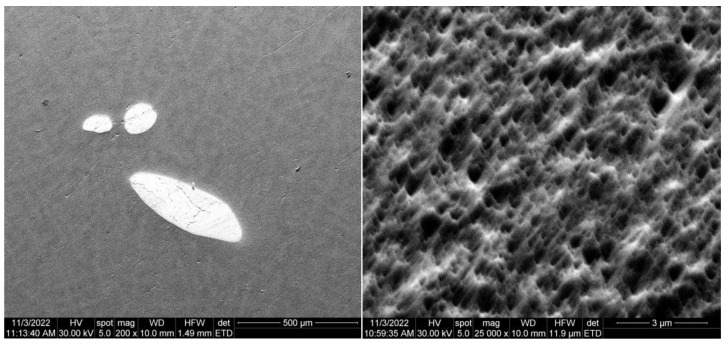
SEM images of a micro-zone showing tungsten accumulation (**left**) and of the corroded area (**right**) of Ti19Mo10W alloy (Code 4).

Another cause is the method of obtaining Ti-Mo-W alloys by melting in the electric arc furnace, which does not offer a good distribution of tungsten in the alloy, and results in the appearance of areas with agglomerations (Figure 12left). To limit this impediment, the use of other methods of obtaining alloys (mechanical alloying and sintering) is being carried out [26].

**Figure 13:**
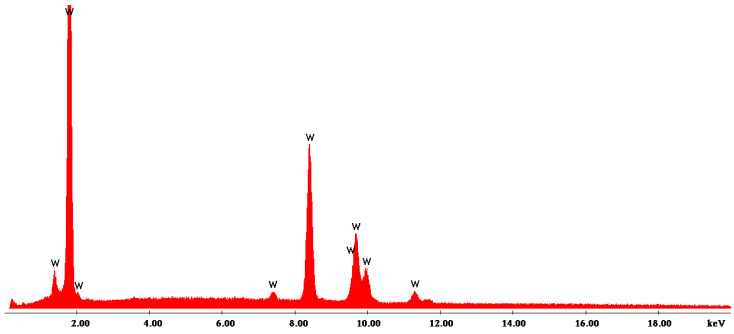
EDS characterization of micro-zone showing tungsten accumulation from Ti19Mo10W alloy (Code 4).

For the accumulation presented in Figure 12, the EDX analysis was carried out, which highlights the presence of Tungsten, as seen in Figure 13.

A better corrosion behavior of a material is highlighted through high polarization resistance (*R_p_*). Therefore, all four Ti alloys have lower values than the value of the reference alloy. Among these, the Ti19Mo7W alloy stands out with a value of 668.69 kΩ × cm^2^.

Comparing the values of the electrochemical parameters corresponding to the alloys investigated from the point of view of corrosion resistance in SBF, it can be concluded that the Ti19Mo7W alloy (Code 1) stands out for the lowest value of the corrosion current density and the lowest corrosion rate.

## 4. Conclusions

Designing and developing titanium alloys for medical implants are necessary to understand the corrosion behavior in biological solutions similarly to the human body environment. The Ti-19Mo-xW (x = 7, 8, 9, 10 wt%) system of alloys was investigated regarding the influence of different contents of tungsten on the corrosion behavior in simulated body fluid (SBF) by means of the linear polarization technique, and correlation with the values of $\bar{Bo}$ and $\bar{Md}$ were made.

Even if the content of Mo reaches 19% and W reaches 10%, the calculated values of $\bar{Bo}$ and $\bar{Md}$ are very close to the values calculated for the Ti-15Mo-xW alloys (x = 1 to 11) previously studied, demonstrating that the alloys researched in the work are currently in the stable beta range. The Mo/W weight ratio in Ti-Mo-W alloys should be maintained close to 3. In the case of Ti-Mo-W alloys, the chemical composition can vary within wide limits if the $\bar{Bo}$ and $\bar{Md}$ values calculated based on the chemical composition are found in the beta Ti domains. Extending the range of beta titanium alloys to a chemical composition of Ti-19Mo-xW, it was found that the concentration of Mo can increase to 19%, only on the condition that the concentration of W is maintained at the lower limit of 7%, in order that the degree of corrosion is minimal. We can conclude that, with the increase in W content from 7 to 10 wt%, the value of $\bar{Bo}$ increases from 2.8281 to 2.8315. The corrosion resistance of alloy samples is dependent on the composition. Based on the results obtained from the corrosion process, the Ti-19Mo-xW alloys prove to be corrosion resistant. Comparing the values of the electrochemical parameters corresponding to the investigated alloys in terms of corrosion resistance in SBF, sample 1—Ti19Mo7W (in which the $\bar{Bo}$ is the lowest—2.8281) has the smallest values of the corrosion current density and corrosion rate. 

## Figures and Tables

**Table 1:** The chemical compositions of pure metals used in the experiment research.

Element	Ti	Mo	W	O	N	C	H	Fe	P	Ni	Al	Si	Ca	Mg	Others
Titanium	99.095	-	-	0.18	0.03	0.08	0.015	0.2	-	-	-	-	-	-	0.4
Molybdenum	-	99.98	-	0.003	0.001	0.005	-	0.005	0.001	0.002	0.002	0.003	0.002	0.001	-
Tungsten	-	0.10	99.87	0.003	0.003	0.005	-	0.005	0.001	0.003	0.002	0.003	0.003	0.002	-

**Table 2:** The elemental analysis of obtained alloys.

Code	Alloy Type	Elemental Analysis, wt%				
		W	Mo	Al	V	Ti
1	Ti19Mo7W	7.17	19.21	-	-	73.62
2	Ti19Mo8W	8.32	19.18	-	-	72.50
3	Ti19Mo9W	9.06	19.37	-	-	71.57
4	Ti19Mo10W	9.79	19.11	-	-	71.10
5	Ti6Al4V	-	-	5.92	4.27	89.81

**Table 3:** The calculated values of $\bar{Bo}$ and $\bar{Md}$.

Code	Alloy Type	Parameters	
		$\bar{Bo}$	$\bar{Md}$
1	Ti19Mo7W	2.82810851	2.3840165
2	Ti19Mo8W	2.8296288	2.38214513
3	Ti19Mo9W	2.83101514	2.38023114
4	Ti19Mo10W	2.83155687	2.37980337

**Table 4:** Values of the main electrochemical parameters determined in the corrosion process.

Code	Sample	*E_cor_* (mV)	*i_cor_* (nA/cm^2^)	*β_c_* (mV)	*β_a_* (mV)	*R_p_* (kΩ × cm^2^)	*CR* (µm/Year)
1	Ti19Mo7W	−333.1	29.214	48.87	557.58	668.69	0.306
2	Ti19Mo8W	−282.6	51.987	168.78	101.54	530.22	0.54
3	Ti19Mo9W	−293.2	62.584	145.21	148.17	509.48	0.647
4	Ti19Mo10W	−272.4	98.861	210.47	227.97	481.28	1.01
5	Ti6Al4V	−186.2	35.415	118.64	189.75	896.18	0.322

## Data Availability

Not applicable.
